# Regulation of Absorption and Emission in a Protein/Fluorophore Complex

**DOI:** 10.1021/acschembio.4c00125

**Published:** 2024-07-24

**Authors:** Elizabeth M. Santos, Ishita Chandra, Zahra Assar, Wei Sheng, Alireza Ghanbarpour, Courtney Bingham, Chrysoula Vasileiou, James H. Geiger, Babak Borhan

**Affiliations:** Department of Chemistry, Michigan State University, East Lansing, Michigan 48824, United States

## Abstract

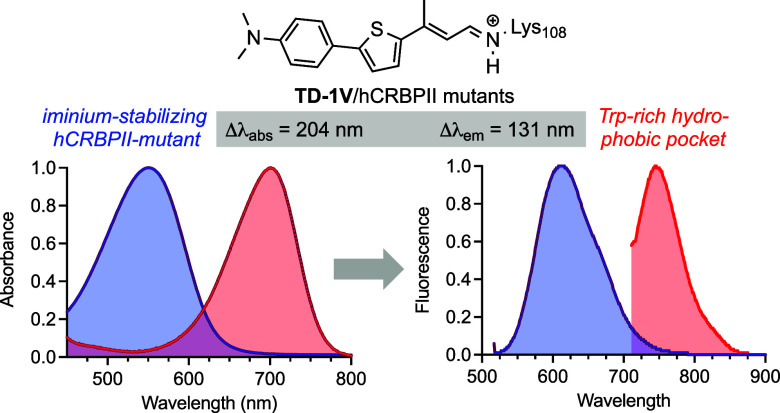

Human cellular retinol binding protein II (hCRBPII) was used as a protein engineering platform to rationally regulate absorptive and emissive properties of a covalently bound fluorogenic dye. We demonstrate the binding of a thio-dapoxyl analog via formation of a protonated imine between an active site lysine residue and the chromophore’s aldehyde. Rational manipulation of the electrostatics of the binding pocket results in a 204 nm shift in absorption and a 131 nm shift in emission. The protein is readily expressed in mammalian systems and binds with exogenously delivered fluorophore as demonstrated by live-cell imaging experiments.

## Introduction

The ability to control the emission properties of fluorophores is crucial in the development of biosensors, imaging probes, fluorescence microscopy, and other optical tools for use in biological systems.^1−4^ In the past 20 years, several innovative labeling strategies have emerged for cellular imaging, blending the genetic precision of proteins with the varied photophysical properties of small-molecule fluorophores. These include FlAsH,^5^ enzyme-based “self-labeling tags” (SNAP-, CLIP-, and Halo-Tag)^6−15^ and electrophilic ligand–receptor pairs (coumarin–photoactive yellow protein, PYP).^16,17^ Self-labeling systems like SNAP-, CLIP-, or Halo-tag have harnessed the flexibility of chemically synthesized fluorophores to create a range of fluorogenic, far- and infrared fluorophores. These advancements cater to the needs of super-resolution microscopy techniques and single molecule studies, offering enhanced versatility and precision in labeling strategies.^10,11,18,19^

On the other hand, systems relying on a noncovalent interaction between the fluorophore and the protein tag offer additional avenues for innovative labeling protocols with the possibilities to label proteins in a fully reversible fashion. Examples of the latter are genetically encodable Fluorogen-Activating Proteins (FAPs) that become fluorescent by immobilization of fluorogenic molecular rotors, or by binding modified thiazole orange (TO) and malachite green (MG).^13,20,21^ At the same time, naturally occurring photoreactive protein-chromophore complexes play an essential role in various biological processes such as vision, phototaxis, and photosynthesis.^22−26^ The effectiveness of these systems hinges on their ability to alter spectroscopic properties via interactions between proteins and chromophores, fluorophores or fluorogenic molecules.

Complexation of a protein with a fluorogenic molecule provides numerous avenues for achieving regulation in emission. Spectral tuning in certain cases is accomplished by structurally altering the chromophore,^27,28^ whereas in others, it results from altering interactions between the protein and chromophore.^29−32^ These include complexation with proteins or other biomolecules, pH and solvent effects.^32−35^ Regarding the latter, we were drawn to the advancements in solvatochromic dyes, which have emerged as a new class of fluorescent probes, as a potential solution to regulate emission wavelength in protein/fluorophore complexes. The term solvatochromism is used to describe the marked change in wavelength of an absorption and/or emission band that results from changing the polarity of the solvent.^36,37^ Often, this is the result of having longer-lived excited states that can affect solvent reorientation in a manner to stabilize the developing polarity. As such, the fluorescent properties of solvatochromic fluorophores can be greatly affected in response to changes in the polarity or hydrogen bonding ability of the solvent. The necessary feature for solvatochromism is for the dipole moment of the fluorophore’s excited state to vary considerably from the dipole moment of its ground state. Thus, a relatively long-lived excited state can be affected by the polarity of the solvent, given the time required for solvent reorganization is available.

Our interest in this area was piqued by our previous successes in controlling the absorptive properties of protein-embedded chromophores in reengineered human Cellular Retinol Binding Protein II (hCRBPII).^31,38^ hCRBPII, a member of the Intracellular Lipid Binding Protein family (iLBP), presents an advantageous platform for protein redesign since it is tolerant to multiple mutations without loss of structural integrity, while possessing a large binding cavity that accommodates structurally diverse ligands.^32,39−42^ We have previously shown that the absorption maximum of all-*trans*-retinal bound as a Schiff base to an active-site lysine residue in hCRBPII can be rationally regulated over 220 nm through electrostatic perturbations.^31^ We surmised that a similar regulation in emissive properties of a bound fluorophore would not only provide a platform for multicolor imaging (use of the same chromophore with different mutants with different fluorescence) but also enable practical no-wash protocols by shifting the emission wavelength of the bound fluorophore away from that of the unbound material (background). The studies in this manuscript reveal two key points: altering the binding cavity’s polarity to achieve solvatochromism is incompatible with protein folding, at least with this family of proteins, which sequester hydrophobic elements internally; and second the emission wavelength of a fluorophore can be shifted as a result of regulating its absorption wavelength by introducing specific interactions that influence protein/ligand interactions. These insights underscore the challenges in designing effective fluorogenic probes.

## Results and Discussion

**Figure 1:**
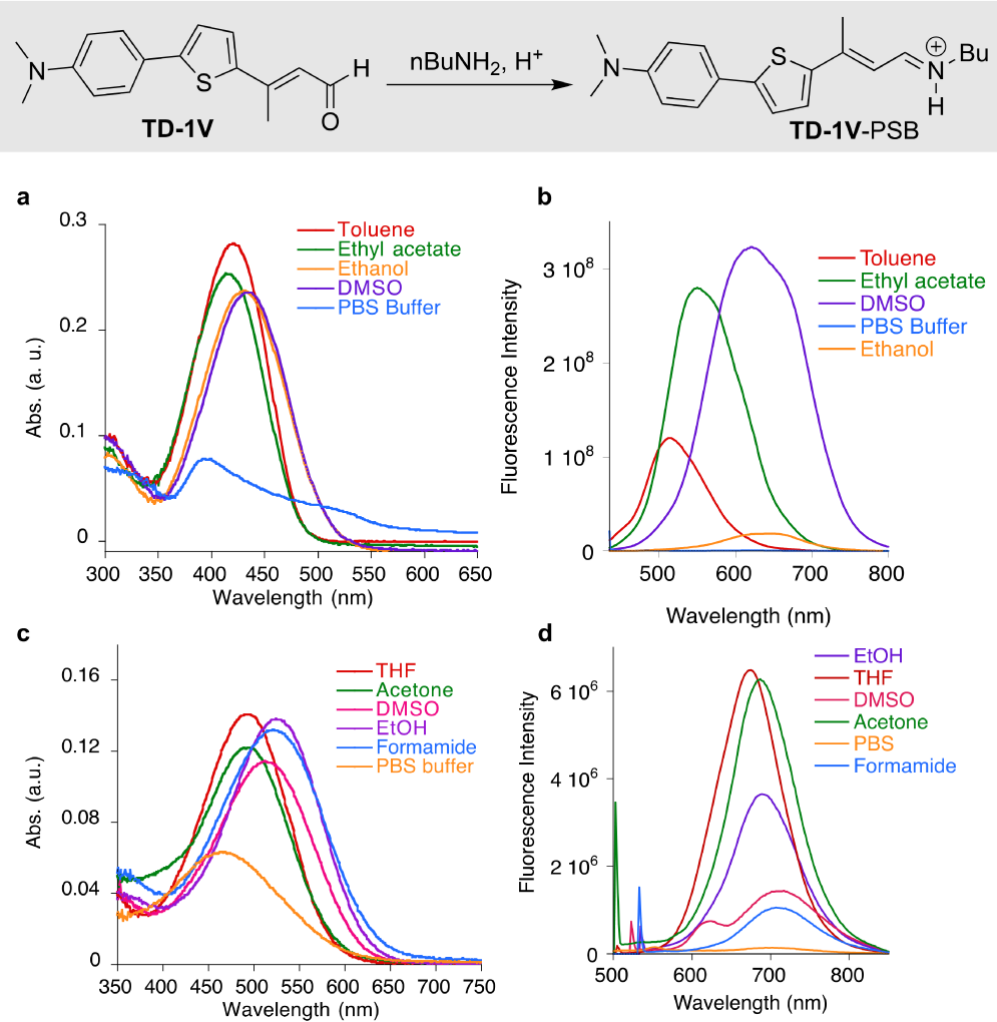
Spectroscopic properties of the free aldehyde **TD-1V** and its corresponding PSB formed with *n*-butyl amine in a variety of solvents. (a) Absorbance and; (b) emission of the free aldehyde; (c) absorbance; and (d) emission of the **TD-1V**-PSB with *n*-butyl amine. Note, the emission values for the PSB in THF were 10 times larger than that shown on the plot.

Previously, we showed the utility of human cellular retinol-binding protein II (hCRBPII) mutants as a fluorophoric tag, wherein an active site lysine residue (Q108K) reacts with synthetic aldehydic chromophores, giving rise to the formation of either an imine (Schiff base, SB) or an iminium (protonated SB, PSB).^31,41,43,44^ We have also recently reported on the use of dapoxyl dye analogs as fluorogenic partners for reengineered hCRBPII mutants to achieve Excited State Proton Transfer (ESPT), yielding large Stokes shift of the protein/fluorophore complex.^41^ The selection of dapoxyl and its structurally related analogs was informed by their well-known solvatochromic characteristics, as well as their potential for generating an intramolecular charge transfer system (ICT) upon PSB formation.^45,46^ Interestingly, **TD**-**1V** (Thio Dapoxyl with 1 vinyl appendage) the dye used in our ESPT studies,^47−49^ shows little change in its absorption maxima when dissolved in different solvents (Figures 1a,c and Figure S2 for a pictorial representation of changes as a function of the solvent’s ET_30_ value). Nonetheless, its emission red-shifts more than 140 nm in ethanol as compared to toluene (Figure 1b). Notably, the fluorophore in PBS buffer is essentially nonfluorescent, presumably due to the formation of aggregates. The nonfluorescent nature of the free aldehyde in PBS buffer is advantageous since it leads to minimal background fluorescence for live-cell imaging, thus improving the signal-to-noise ratio in fluorescence-based assays. The former characteristics of **TD-1V** made it an appealing choice to explore as a candidate for emission wavelength regulation in protein/fluorophore complexes.

**TD-1V**-PSB, obtained by reacting **TD-1V** with *n*-BuNH_2_, was investigated as a surrogate for the iminium formed with **TD-1V** and the hCRBPII mutants (Figures S1 and S2). As can be seen from Figure 1d, it shows less solvatochromism for emission as compared to **TD-1V**, although its absorption is more sensitive to the nature of the solvent.

We began our investigations with the goal of engineering a solvatochromic protein/fluorophore complex. In our initial study, we meticulously examined over 100 mutation combinations of 10 different residues that lined the interior of the protein cavity in order to alter the polarity of the binding pocket. Nonetheless, it became glaringly apparent that our efforts to modify the global polarity of the protein’s binding pocket had an unintended consequence—we observed a substantial reduction in the expression of soluble protein, concomitant with the production of insoluble inclusion bodies (see Table S7 for a list of mutants). In retrospect, this is not surprising since altering the global polarity of the binding pocket estimated to mimic the dielectric constant of cyclohexane and toluene (2–4)^50,51^ to match that of a polar solvent such as ethanol or acetonitrile (25–35)^52^ would require significant changes to the interior of the protein. Presumably, introduction of hydrophilic residues at the level required within the interior of the protein, which is often hydrophobic, leads to protein destabilization, causing protein unfolding to expose the interior. Hence, we abandoned efforts to mimic global solvent polarity in a protein environment to achieve solvatochromicity, as it proved not to be feasible, even in the mutationally resilient hCRBPII template.

With the failure to make global changes to the polarity of the binding pocket, two points were critical in our efforts to achieve regulation in emission. First, our previous efforts in achieving changes in the absorption wavelength of retinylidene bound hCRBPII mutants was successful through localized changes in electrostatics by the introduction of specific amino acid interactions with the bound polyene, without radically changing the overall polarity of the binding pocket.^31^ Second, **TD-1V**-PSB (with *n*-BuNH_2_) was less sensitive to solvent polarity as compared to **TD-1V** (less solvatochromic), however, it exhibited the largest change in absorption and emission between protic and aprotic solvents,^41^ suggesting that local interactions that can be engineered in a protein environment might be able to yield reasonable levels of wavelength regulation.

With the goal of using **TD-1V** as a fluorogenic dye, we began to screen hCRBPII mutants that bound the aldehyde as a PSB via an active-site lysine residue. Changes in the emission wavelength were planned as the result of alterations to the protein sequence that would result in hypso- and bathochromic shifts in the absorption of the protein/fluorophore complex. We had previously shown with hCRBPII/retinal complexes that an even distribution of electrostatic potential across the entire chromophore is essential for maximal bathochromic shifting, while localization of positive charge on the iminium by placement of carboxylate residues nearby led to blueshift of absorption.^31^ Toward red shifting of the absorption, and consequently its emission, we sought to eliminate polarity through two distinct approaches: 1) removal of the more polar residues in the binding pocket nearest the chromophore and 2) removal of water molecules that either directly or indirectly interact with the iminium. We envisioned that the removal of negative electrostatic potential in the PSB region would encourage positive charge delocalization.

The study was initiated with the double mutant Q108K:K40L (KL/**M1**) hCRBPII. These two mutations were previously shown necessary to facilitate binding of an aldehydic chromophore. Q108K functions as the active-site lysine and forms a Schiff base with the aldehyde, whereas the K40L mutation is assumed to eliminate interactions between Q108K and Lys40, an interaction that could otherwise impede imine formation, and is also required to promote Schiff base protonation. With this template in hand, mutagenesis of polar residues in proximity to the chromophore were pursued (Thr51, Thr53, Gln4, Gln38, and Arg58, Figure S3). Systematic changes, as shown in Table 1, led to red-shift of the protein/**TD-1V** complex from 580 to 608 nm. Residues closer to the iminium (Gln4, and Thr51) led to a larger redshift than those situated further away (Thr53 and Gln38, Table 1). Significant insight into wavelength tuning could be derived from our previous studies of retinal-bound hCRBPII,^29,31^ with many, though not all, of the same modifications leading to red shifting with the system described here.

**Table 1:** Residues Mutated in Order to Remove Polarity in the Vicinity of the Chromophore

mutant	protein^a^	λ_abs_ (nm)	λ_em_ (nm)	Φ^b^
**M1**	Q108K:K40L (KL)	580	674	nd
**M2**	KL:T51V	608	690	0.12
**M3**	KL:T53V	597	690	0.13
**M4**	KL:T53A	585	677	0.11
**M5**	KL:T53S	587	682	0.08
**M6**	KL:Q38F	588	680	0.13
**M7**	KL:Q4F	607	691	nd
**M8**	KL:T51V:T53S	627	697	0.15

a 20 μM protein and 0.5 equiv of TD-1V.

b Absolute quantum yield was measured on a Quantaurus-QY. Not detected (nd).

The structurally conservative substitution of Thr51 with valine, yielding the Q108K:K40L:T51V triple mutant (**M2**) in hCRBPII resulted in a remarkable 28 nm red-shift in absorption and a subsequent 16 nm red-shift in emission as compared to the parent template. Beyond its role in shifting the wavelength, the T51V mutation greatly reduced the previously observed domain swapped dimerization of the protein,^53−55^ irrespective of most changes at other residues. Notably, hCRBPII/**TD-1V** complexes exhibit markedly distinct spectroscopic properties of their monomeric and domain-swapped dimeric variants.

Subsequently, our investigation aimed to alter the polarity of the Thr53 position, which makes a water mediated hydrogen bond with Thr51 in crystal structures where both residues are maintained (Figure S4). Substitution with the isosteric valine (**M3**) red-shifted both absorption and emission by 17 and 16 nm, respectively. The T53A mutation also red-shifted absorption and emission, but far more modestly (5 and 3 nm respectively, compare **M4** to **M1**). The T53S mutation (**M5)** red-shifted both absorption and emission by 7 and 8 nm, respectively, even though serine has similar polarity as threonine. Moreover, the addition of tryptophan at Arg38 (**M6**) also red-shifted absorption and emission.

Gratifyingly, the red shifting properties of T51V and T53S to **M1** were additive, resulting in 47 and 23 nm, absorption and emission red shifts, respectively (**M8**, Table 1). Thus, the **M8** template (Q108 K:K40L:T51V:T53S) was retained for further protein engineering. Although the Q4F mutation (**M7**) showed substantial, red-shifted absorption and emission, this was observable only after acidification of the media, as the imine bond formed at physiological pH was not protonated. Apparently the Q4F modification led to a depressed p*K*_a_ of the iminium, an occurrence that we had noted before in our prior protein engineering efforts.^41^ For this reason, we decided to include it only after we had identified all of the critical residues, with the caveat that there might be a need for further sequence adjustments to achieve a ground state protonated species (PSB).

We next turned our attention to the entrance to the binding pocket because our previous studies with retinal showed that introduction of aromatic residues in this region leads to the closure of the binding pocket and results in substantial bathochromic shifts. We first focused on the Arg58 position, introducing aromatic residues (**M9**-**M12**) to the parent tetramutant **M8** (Table 2). However, results were disappointing with most mutations resulting in modest hypsochromic wavelength shifts. Of the four aromatic residues only **M12** with the R58H mutation led to a bathochromic shift, possibly due to its ability to support hydrogen bonding interactions between the positively charged histidine side chain and the *N,N*-dimethyl amino moiety of the ligand. Unfortunately, crystallization of Q108K:K40L:T51V:T53S:R58H (**M12**) was not fruitful. Nonetheless, we were able to successfully obtain a crystal structure of **M11**, which guided further engineering efforts.

**Table 2:** Mutation at R58 to Encapsulate the Binding Pocket

mutant	protein^a^	λ_abs_ (nm)	λ_em_ (nm)	Φ^b^
**M8**	KL:T51V:T53S	627	697	0.12
**M9**	**M8**:R58F	617	691	0.11
**M10**	**M8**:R58Y	622	691	0.08
**M11**	**M8**:R58W	623	697	0.12
**M12**	**M8**:R58H	636	703	0.14
**M13**	**M8**:R58H:Y19W	636	700	0.15
**M14**	**M8**:R58W:Y19W	653	719	0.10

a 20 μM protein and 0.5 equiv of TD-1V.

b Absolute quantum yield was measured on a Quantaurus-QY.

**Figure 2:**
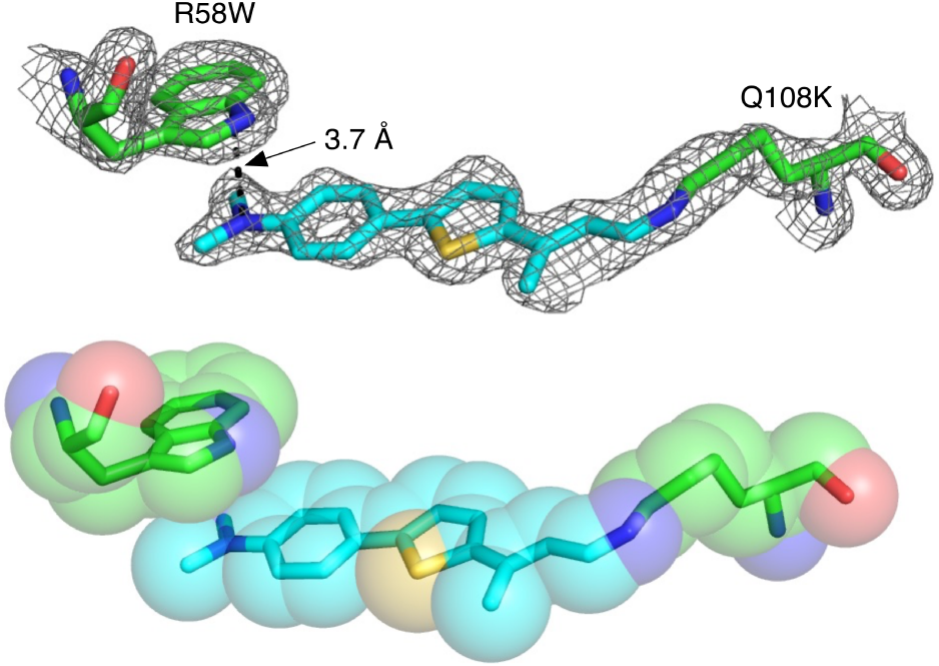
Crystal structure of **M11**/**TD-1V** and space-filling representation. Electron density is shown at 1σ.

As seen in Figure 2, the tryptophan at position 58 does not sequester the binding pocket; instead, it flips inside, where it makes a cation - π interaction with the diethyl-amino group of **TD-1V** (3.7 Å). Additionally, comparison of the **M10** and **M11** structures shows that introduction of Trp58 results in an almost 90° rotation of the entire chromophore, altering the position and therefore interactions of the imine at the opposite end (Figure S5). In short, a number of structural factors explain why, in contrast to most other mutations, R58W does not give the substantial red shifting with **TD**-**1V** in comparison to the bathochromic shift observed with retinal-bound complexes (**M11**, Table 2).^31^

**Figure 3:**
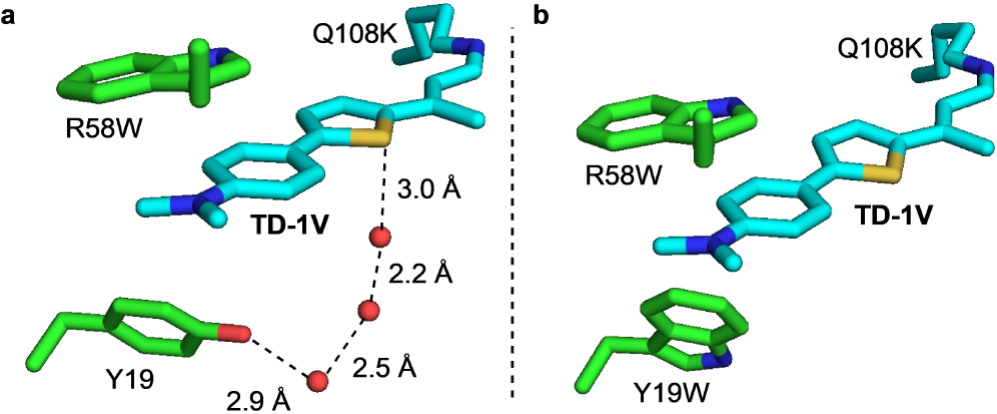
(a) Crystal structure of **M11**/**TD-1V**, highlighting the water mediated network from Y19 to **TD-1V**. (b). Crystal structure of **M14**/**TD-1V** shows this water network is abolished.

A conserved water network connecting the hydroxyl group of Tyr19 and the thiophene sulfur atom of **TD-1V** was observed in the **M11** crystal structure (Figure 3a). This observation marked Tyr19 as a residue for exchange into one that could disrupt this organized water network. Although incorporation of the Y19W mutation with R58H did not alter the spectroscopic characteristics of the protein/fluorophore complex (**M13**, Table 2), surprisingly, when coupled with R58W, it led to a 30 and 22 nm red-shift in absorption and emission, respectively (Table 2, **M11** vs **M14**).

Gratifyingly, the crystal structure of **M14**/**TD-1V** was obtained and showed that substitution of tryptophan for Tyr19 did indeed disrupt the water network observed in the **M11**/**TD-1V** crystal structure (Figure 3b). The explanation for why only the combination of Y19W with R58W led to the significant change in spectral shift as opposed to the combination of R58H and Y19W is probably due to the nearly 90° rotation of the chromophore trajectory enforced by its interaction with Trp58 as described previously for **M11** (Figure S5). Exactly the same orientation is seen in the **M14**/**TD-1V** crystal structure, confirming the key and exclusive role Trp58 plays in causing the rotation of **TD-1V** in the binding pocket (Figure S6). Collectively, this points to the fact that the result of single mutations is not additive when significant changes in chromophore orientation result. For example, neither R58W nor Y19W are individual “winners”, but together they lead to a substantial effect. With the changes thus far, **M14**/**TD-1V** complex exhibits a bathochromic shift in absorption and emission (Δ_abs_ = 73 nm, Δ_em_ = 45 nm) relative to its parent mutant complex (**M1**/**TD-1V**).

### Encapsulating the Binding Cavity for Further Red-Shifting

**Figure 4:**
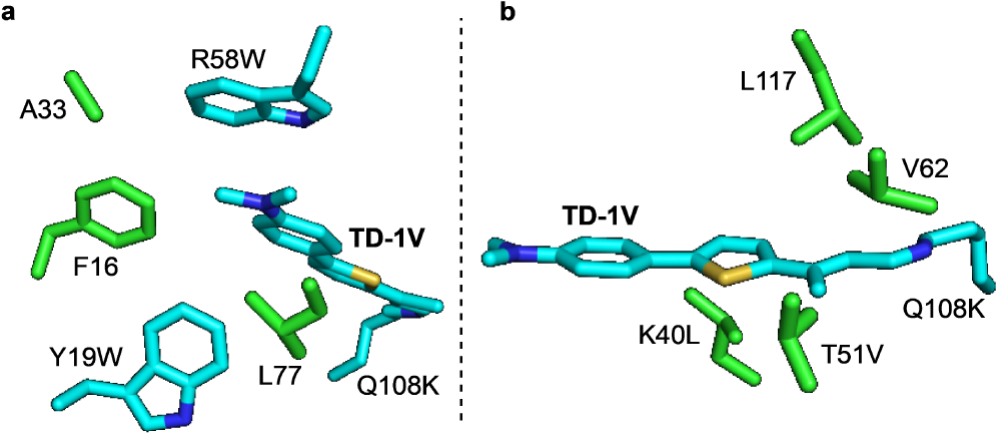
(a) Residues mutated (shown in green) to encapsulate the binding cavity. Crystal structure is of **M14**/**TD-1V**. (b) Positions for introducing an acidic reside to blue shift wavelength based on the crystal structure of **M11**/**TD-1V**.

Efforts to further red shift the **M14** hexamutant turned once again to further enclosing the binding pocket. Aromatic residues (in particular Trp) were not only introduced to reduce the ingress of water into the binding pocket, but also lead to a tighter packing of the chromophore to reduce vibrational freedom, and potentially increase quantum yield. The polarizability of aromatic residues could also yield further red-shift of the protein/fluorophore complex by stabilizing the cationic charge along the polyene. For this purpose, mutations of Ala33, Leu77, and Phe16 were introduced within the **M14** hexamutant. Ala33 and Leu77, located at the opening of the protein cavity, were predicted to further sequester the binding pocket, while Phe16, found in the interior of the protein, was thought to lie close to the bound fluorophore (Figure 4a).

Tryptophan mutations were singly made at three positions (Phe16, Ala33 and Leu77) on the **M14** template, with the latter two located at or near the mouth of the binding pocket, in an attempt to further isolate **TD-1V** from the bulk aqueous solvent (Figure S7). Of these, the A33W mutation provided the largest red shift in both absorbance and emission (**M15**-**M17**, Table 3). The F16W mutation was not tolerated but the F16Y variant gave a significant red shift. Interestingly, inclusion of both the Q4W and A33W mutations (**M18**) led to the most red-shifted absorption and emission (λ_abs_ = 705 nm, λ_em_ = 744 nm) in the series. This is likely the result of the elimination of the water-mediated interaction between Gln4 and the iminium, resulting in enhanced delocalization of the positive charge in the excited state, similar to that seen with retinal bound complexes (Figure S8). Introduction of tryptophans at other locations along the polyene (**M19**-**M21**, Table 3) had little effect on the spectroscopic behavior of the protein/**TD-1V** complex.

**Table 3:** Residues Mutated in Order to Tightly Pack the Chromophore

mutant	protein^a^	λ_abs_ (nm)	λ_em_ (nm)	Φ^b^
**M14**	**M8**:R58W:Y19W	653	719	0.10
**M15**	**M14**:A33W	666	724	0.19
**M16**	**M14**:L77W	658	711	0.10
**M17**	**M15**:F16Y	664	726	0.05
**M18**	**M15**:Q4W	705	744	0.10
**M19**	**M15**:Y60W	661	718	0.10
**M20**	**M15**:S76W	665	721	0.19
**M21**	**M15**:L119W	665	729	nd
**M22**	**M14**:L117E	563	673	0.31
**M23**	**M14**:L117D	525	618	0.30

a 20 μM protein and 0.5 equiv of TD-1V.

b Absolute quantum yield was measured on a Quantaurus-QY. Not detected (nd).

### Blue-Shifted Protein Complex by Stabilizing the Iminium Cation

Introduction of acidic residues near the iminium should localize the positive charge on the iminium, resulting in a blue-shift of the absorption/emission of the protein/**TD-1V** complex (Figure 4b). To this end, K40E is an obvious choice due to its close proximity to the iminium. However, mutants containing the latter change did not yield a PSB upon complexation with **TD-1V** because of their depressed p*K*_a_. Acidification of the buffer to protonate the imine led to the precipitation of the protein. Our prior experience with these proteins also informed our decision to maintain the K40L mutant for stability concerns, and thus we pivoted our strategy by incorporating acidic residues into the previously red-shifted template **M14**. Subsequently, we mutated Leu117 to both Asp and Glu, resulting in a distinct, blue-shifted iminium (Table 3, **M22**–**M23**). This established a route by which one could blue-shift the protein complex. The protein could be further blue-shifted by incorporation of two acidic residues close to the iminium (see **M32** λ_abs_ = 501 nm, λ_em_ = 613 nm, Table S8), although this required further modifications of the protein sequence to support the formation of an iminium. In our study, predicting the quantum yields of different mutants proved challenging. Most mutants, irrespective of their sequence, exhibited quantum yields in the range of 10–15%. Nonetheless, we did observe a discernible pattern in the quantum yields of mutants featuring acidic residues proximal to the iminium moiety. Interestingly, these mutants exhibited higher quantum yields, most greater than 30%. We attribute this to the propensity for salt-bridge formation between acidic residues and the iminium, which putatively would lead to increased rigidity of the bound chromophore, and thus reduced vibrational freedom, which in turn would reduce nonradiative relaxation pathways.

### Emission is Linearly Correlated to Absorbance

**Figure 5:**
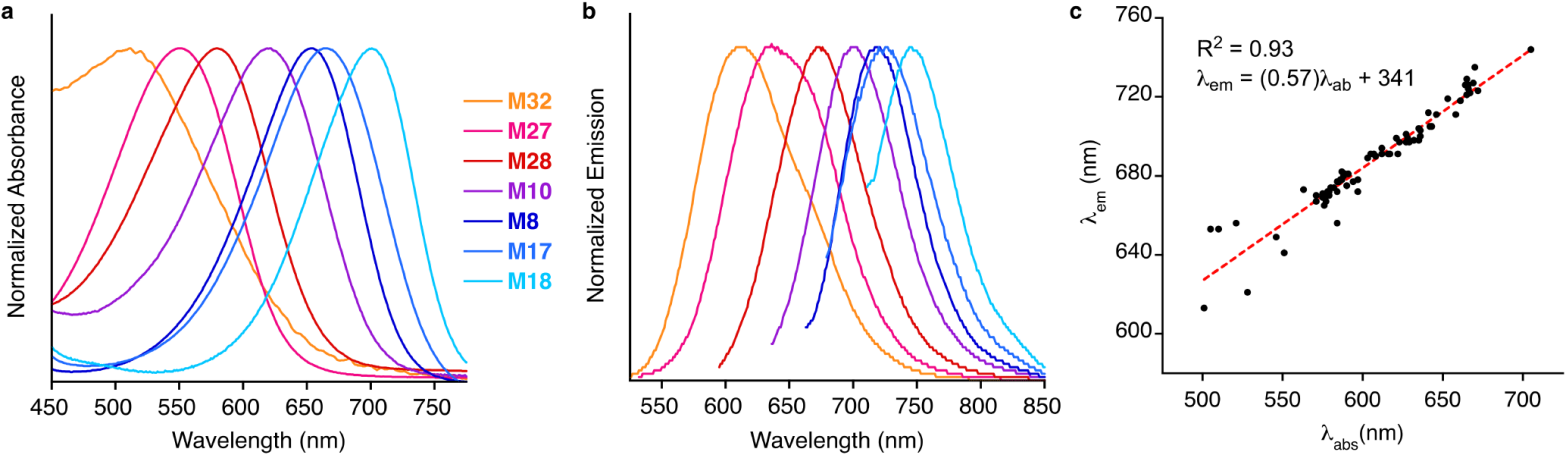
(a) Range of absorption and (b) emission achieved in this study by coupling hCRBPII monomers with **TD-1V**. Mutants from left to right are **M32**, **M27**, **M28**, **M10**, **M8**, **M17**, and **M18** (**M25**–**M34**, Table S8); (c) plot of emission versus absorbance for the approximately 80 hCRBPII/**TD-1V** complexes examined in this study yields a linear fit, with R^2^ = 0.93.

Figure 5a,b depicts select hCRBPII mutants coupled with **TD-1V**, illustrating wavelength regulation of the protein/fluorophore complex, spanning absorption maxima from 501 to 705 nm and emission maxima from 613 to 744 nm. This is equivalent to regulation over 204 nm in absorption and 131 nm in emission, covering both the red and far-red fluorescence wavelength regimes. Figure 5c plots the emission vs absorbance for all protein/**TD-1V** complexes examined for this study. A linear correlation with an R^2^ = 0.93 and a slope of 0.57 indicates that approximately a 1 nm red-shift is gained in emission for a 2 nm change in absorption. Note, in contrast to the iminium in the protein, **TD-1V**-PSB formed with *n*-BuNH_2_ showed little shift in absorption maxima in different solvents (Figure 1c). Nonetheless, the emission of the latter PSB did exhibit a substantial variation, spanning over 140 nm, from the nonpolar toluene to the more polar ethanol. Thus, although not through solvatochromism, we were able to substantially expand the amount of emission regulation possible in the protein environment relative to solvent, through a different mechanism (wavelength regulation of the absorption) as compared to what is observed in solvents of different polarity.

### Live-Cell Imaging

Proof-of-concept live-cell imaging was carried out with **M22** (Q108K:K40L:T51 V:T53S:R58W:Y19W:L117E) heptamutant since it possessed a high quantum yield upon complexation with **TD-1V** (31%), a high p*K*_a_ of its iminium (11.2), and a reasonable *t*_1/2_ for binding (82 min, second-order kinetics; measured at 23 °C with 20 μM protein and 0.5 equiv **TD-1V** at pH ∼ 7, see Figure S9). Its absorption and emission maxima (λ_abs_ = 563 nm, λ _em_ = 673 nm) are also well aligned with lasers available on commercial confocal microscopes. Additionally, it is expressed solely in its monomeric form. It is important to note that some hCRBPII mutants can express as mixtures of monomers and domain-swapped dimers. In fact, we have previously examined structural factors that can promote either form,^53−55^ however the data presented for all mutants in this manuscript are from the monomeric form since the proteins were purified via size exclusion chromatography. Nonetheless, for mammalian expression, we chose the variant that shows little to no expression of the domain-swapped dimer to avoid complications that could arise from mixtures. It is important to note that with most protein/chromophore complexes reported here, it is not possible to report an accurate extinction coefficient since the complex exists in an equilibrium of the SB and PSB forms. Yet, with a select few mutants that have high iminium p*K*_a_ one can assume the concentration of the PSB is that of the protein, and thus, extinction coefficients can be estimated. Protein complexes with high p*K*_a_ of their iminium exhibited extinction coefficients of ∼50,000 M^–1^cm^–1^.

**Figure 6:**
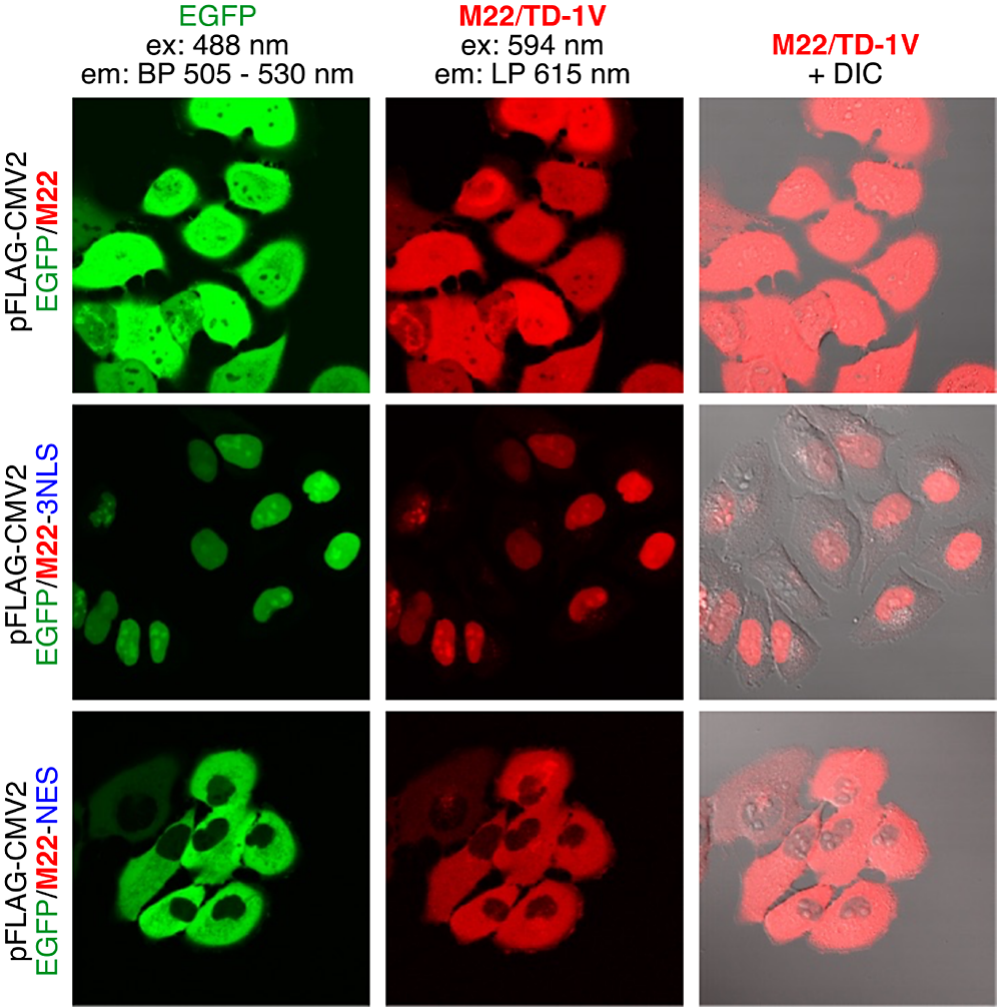
Labeling of HeLa cells expressing **M22**-EGFP, **M22**-EGFP-3NLS and **M22**-EGFP-NES with 10 μM **TD-1V** (incubated at 37 °C for 1 h). NLS = nuclear localization sequence and NES = nuclear export signal.

**M22** was cloned into the pFlag-CMV2 vector containing EGFP, with and without localization peptides (whole cell: hCRBPII-EGFP, nucleus: hCRBPII-EGFP-3NLS, and extranuclear space: hCRBPII-EGFP-NES). Imaging was performed by incubating HeLa cells with 10 μM **TD-1V** for 1 h at 37 °C. The cells were then washed with PBS buffer two times before imaging. In each instance, the successful expression of the fusion protein was confirmed by the observation of the control GFP upon excitation at 488 nm (Figure 6, green channel). Upon excitation at 594 nm, the images obtained from the **M22**/**TD-1V** complex showed remarkable similarity. Importantly, there was no apparent background fluorescence in the red channel, signifying that **TD-1V** specifically labels the intended lysine residue without reacting with off-target amines. As depicted in Figure 6, whole cell labeling, nuclear localization, and cytosol labeling proceeded with success.

## Conclusion

In summary, our study successfully achieved wavelength tuning by coupling the fluorophore **TD-1V** with various hCRBPII mutants. The structure-based rational design strategy used here were in many cases similar to that used to tune the wavelength of retinal, which shows that our ability to subtly tune the electrostatic environment will give similar results, even with a chromophore substantially different in structure, as long as a resonating positive charge is present. Thus, principles established here and in our previous studies are likely translatable to other protein/chromophore systems, providing a “toolbox” for protein-based chromophore tuning. Protein complexes exhibited wavelength regulation in absorption ranging from 501 to 705 nm, while in emission the maxima spanned from 613 to 744 nm. This equates to a regulation over 204 nm in absorption and 131 nm in emission, effectively covering both the red and far-red fluorescence wavelength regimes. This also represents a potential advantage of our fluorescent protein system that enables modulating absorption or emission of a single fluorophore similar to what has been described recently for Halo-tag variants.^56^ The use of **TD-1V** in live-cell imaging was demonstrated by effectively targeting the nucleus and extranuclear regions, with minimal background fluorescence. The ultimate goal would now be to demonstrate success in multicolor imaging by using two different hCRBPII variants targeted to different intracellular organelles (i.e., one variant targeted to the nucleus and the second targeted to the extra nuclear space), labeled with the same ThioFluor dye **(TD-1V)**, to allow for multiorganelle imaging simultaneously.

## Supporting Information Available

Experimental data including synthesis of the fluorophores, protein expression and purification, UV–vis spectra, crystallization conditions, and X-ray data collection and refinement statics and crystallographic files such as atomic coordinates and structure factors have been deposited in the Protein Data Bank, (PDB ID codes: 8VZX, 8VZY, 8W02, 8VZZ, 8W00) (PDF)

## Abbreviations

QY: quantum yield
FP: fluorescent protein
NIR: near infra-red
ESPT: excited state proton transfer
SB: Schiff base
PSB: protonated Schiff base
hCRBPII: human cellular retinol binding protein II
Å: angstrom
ε: extinction coefficient
EGFP: enhanced GFP
NLS: nuclear localization sequence
PBS: phosphate buffered saline
PDB: protein data bank
NES: nuclear exclusion sequence
Abs: absorption
Em: emission
