# Supplementary material for: Regulation of Absorption and Emission in a Protein/Fluorophore Complex
Source: ACS Chem Biol. 2024 Jul 24;19(8):1725–32. doi: 10.1021/acschembio.4c00125 (PMC11334107; doi:10.1021/acschembio.4c00125)
Supplement: Supplementary file 1 — cb4c00125_si_001.pdf [file cb4c00125_si_001.pdf]

Supporting Information for:

Regulation of Absorption and Emission in a Protein/Fluorophore Complex

Elizabeth M. Santos, Ishita Chandra, Zahra Assar, Wei Sheng, Alireza Ghanbarpour, Courtney Bingham, Chrysoula Vasileiou, James H. Geiger,* Babak Borhan*

Department of Chemistry, Michigan State University, East Lansing MI 48824

A. GENERAL METHODS 3
B. SITE-DIRECTED MUTAGENESIS 3
C. PROTEIN EXPRESSION AND PURIFICATION OF HCRBPII IN PET-17B 7
D. EXTINCTION COEFFICIENT DETERMINATION OF HCRBPII MUTANTS 10
E. UV-VIS MEASUREMENTS OF HCRBPII/CHROMOPHORE COMPLEXES 10
F. PK_A MEASUREMENTS OF HCRBPII/CHROMOPHORES COMPLEXES 10
G. KINETIC MEASUREMENTS OF HCRBPII/CHROMOPHORE PSB FORMATION 11
H. FLUORESCENCE MEASUREMENTS 12
I. QUANTUM YIELD MEASUREMENTS 13
J. CLONING FOR MAMMALIAN EXPRESSION VECTORS 13
K. DNA SEQUENCES OF MAMMALIAN PLASMIDS 15
L. MAMMALIAN CELL CULTURE 18
M. GENERAL CONFOCAL IMAGING METHOD 19
N. SPECTROSCOPIC TABLES OF TD-1V AND ITS BOUND FORM WITH MUTANTS. 19
O. SUPPORTING INFORMATION FIGURES 23
P. PROTEIN CRYSTALLIZATION AND DATA COLLECTION 28
Q. CRYSTALLOGRAPHIC DATA AND REFINEMENT STATISTICS 29
R. REFERENCES 30

A. General Methods

UV/Vis spectroscopy was performed with a Cary 300 Bio UV-Visible Spectrophotometer (Varian) using a 1 cm pathlength (1 mL) quartz microcuvettes (Starna Cells). Fluorescence spectroscopy was performed on a Fluorolog®-3 spectrofluorometer (Jobin Yvon, Horiba Scientific) with a 1 cm pathlength (3.5 mL) quartz cuvettes or a 1 cm pathlength (1 mL) quartz microcuvettes (Starna Cells). All measurements were taken at ambient temperature (23 ± 1 °C) unless specified. **TD-1V** (Thio Dapoxyl with 1 vinyl appendage) was synthesized as previously described.[1] All DNA oligos were synthesized by Integrated DNA Technologies (IDT) on a 25 nmol scale.

B. Site-Directed Mutagenesis

The pET-17b plasmid, containing hCRBPII-Q108K:K40L cloned between NdeI and XhoI, was used as a template for mutagenesis of hCRBPII.[2] Site-directed mutagenesis was conducted via polymerase chain reaction (PCR), with the specified cycling conditions shown in **Table S1**.

Table S1. PCR cycling conditions for site-directed mutagenesis.

	PCR Program	Time (min)
1x	94 °C	3:00
20x	94 °C	0:20
	3 – 5 °C below T_m	0:55
	72 °C	3:30
1x	72 °C	10:00
1x	4 °C	5:00

Reactant	Volume
DNA template	70 ng (x μL)
Forward primer	20 pmol (y μL)
Reverse primer	20 pmol (z μL)
10 mM dNTP	1 μL
50 mM $MgCl_2$	1 μL
DMSO	5 μL
10 x Cloned Pfu Reaction Buffer	5 μL
Pfu Turbo DNA polymerase (2.5 U/μL)	1 μL
dI water	50 μL – x – y – z – 7 μL

The primers used for mutagenesis were ordered from Integrated DNA Technologies (IDT), with melting temperatures (T_m) from approximately 52 °C to 65 °C. The sequences of the forward primers (5' to 3') are listed below. It should be noted that, in all cases, the reverse primer is the reverse complement of the forward primer.

Q4E - GACGAGGGACGAGAATGGAACC

Q4F - GACGAGGGACTTCAATGGAACC

Y19W - CTTTGAGGGCTGGATGAAGGC

R58W - CTAGCACATTCTGGAACTATGATGT

A33W - CGC AAG ATT TGG GTA CGT CTC AC

Q38F - GTACGTCTCACTTTTACGAAGGTTATTGAT

K40L - CTCACTCAGACGCTGGTTATTGATCAAGATGG

K40E - CTCAGACGGAGGTTATTGAT

T51V - GGTGATAACTTCAAGGTAAAAACCACTAGCAC

T53A - CTTCAAGACAAAAGCTACTAGCACATTCCG

T53S - CTTCAAGACAAAATCCACTAGCACATTCCG

T53V - CTTCAAGACAAAAGTCACTAGCACATTCCG

R58W - CTAGCACATTCTGGAACTATGATGTG

R58F - CTAGCACATTCTTCAACTATGATGTG

R58Y - CTAGCACATTCTACAACTATGATGTG

R58H - CTAGCACATTCCACAACTATGATGTG

R58L - CTAGCACATTCCTGAACTATGATGTG

Q38F - GTACGTCTCACTTTTACGAAGGTTATTGAT

K40L - CTCACTCAGACGCTGGTTATTGATCAAGATGG

T51V - GGTGATAACTTCAAGGTAAAAACCACTAGCAC

T51D - GGTGATAACTTCAAGGATAAAACCACTAGCAC

T53A - CTTCAAGACAAAAGCTACTAGCACATTCCG

T53S - CTTCAAGACAAAATCCACTAGCACATTCCG

T53V - CTTCAAGACAAAAGTCACTAGCACATTCCG

R58W - CTAGCACATTCTGGAACTATGATGTG

R58F - CTAGCACATTCTTCAACTATGATGTG

R58Y - CTAGCACATTCTACAACTATGATGTG

R58H - CTAGCACATTCCACAACTATGATGTG

Y60W - GCACATTCTGGAACTGGGATGTGGATTTC

V62E - AACTATGATGAGGATTTCACTGTTGGAGTA

S76W - GTACACAAAGTGGCTGGATAACCG

L77W - GTACACAAAGAGCTGGGATAACCGGCATG

L117E - CAAGCTGTACGACGAGCTGACC

L117D - CAAGCTGTACGAGGAGCTGAC

The crude PCR product was then digested with 20 units DpnI enzyme (New England Biolabs) for one hour at 37 °C. The resulting solution (7 µL) was then added to *E. coli* XL-1 Blue competent cells (Novagen, 100 µL) on ice for 30 minutes. Subsequently, the cells were heat shocked for 30 seconds at 42 °C and then gently spread on a Luria broth (LB) agar plate supplemented with 100 µg/mL ampicillin and 12.5 µg/mL tetracycline. The plate was incubated at 37 °C for approximately 16 hours. A single colony was then inoculated into 10 mL LB media supplemented with 100 µg/mL ampicillin and 12.5 µg/mL tetracycline. LB media was prepared by adding 10 g tryptone, 7 g yeast extract and 5 g NaCl to 1 L deionized water. The media was autoclaved and cooled to room temperature before use. The inoculated culture was shaken at 37 °C for 12 hours. DNA purification was performed using a Promega Wizard Plus SV miniprep DNA purification system (A1330). The concentration of the isolated plasmid was measured via Nandrop; the average concentration was 100 ng/µL. Every sample was sequenced by the Research Technology Support Facility at

Michigan State University, using a primer corresponding to the T7 promoter for all samples in pET-17b plasmid.

C. Protein Expression and Purification of hCRBPII in pET-17b

The target gene (100 ng of DNA for 100 µL cell solution) was added to thawed BL21(DE3) pLysS competent cells (Invitrogen) on ice and incubated for 30 minutes. Subsequently, the cells were heat shocked for 30 seconds at 42 °C and then gently spread on a Luria broth (LB) agar plate supplemented with 100 µg/mL ampicillin and 27 µg/mL chloramphenicol. The plate was incubated at 37 °C for approximately 12 hours.

A single colony was then inoculated into 1 L terrific broth (TB) media supplemented with 100 µg/mL ampicillin and 27 µg/mL chloramphenicol. TB media was prepared by mixing two solutions and autoclaving them separately. The first solution consists of 12 g tryptone, 24 g yeast extract and 4 mL glycerol to 900 mL deionized water. The second solution was prepared by mixing 2.31 g KH_2PO_4 and 12.54 g of K_2HPO_4 in 90 mL deionized water. Before inoculation the solutions were mixed. The inoculated culture was shaken at 37 °C until optical density (OD) at 600 nm was approximately 1; this typically takes eight to nine hours. Overexpression was induced by the addition of isopropyl-β-D-thiogalactopyranoside (IPTG, Gold Biotechnology) at a final concentration of 1 mM. The culture was then shaken at 23 °C for 20 hours.

The cells were harvested by centrifugation (5000 rpm, 10 min, 4 °C) and resuspended in Tris-binding buffer (10 mM Tris, pH = 8.0, 50 mL). The cells were then lysed by sonication (Biologics, Inc, power 60%, 3 min). The solution was again centrifuged to separate the pellet and supernatant (5000 rpm, 30 min, 4 °C). All further protein

purification was also conducted at 4 °C.

The supernatant was then loaded onto a FastQ anion exchange column pre equilibrated with Tris buffer (10 mM Tris, pH = 8.0). After binding of the protein to the FastQ anion exchange resin (GE Healthcare), the column was washed twice with Tris buffer (2 x 50 mL). Lastly the protein was eluted with Tris-elution buffer (10 mM Tris, 200 mM sodium chloride, pH = 8.0, 100 mL). The eluent from the FastQ anion exchange column was then desalted with Tris buffer using an ultrafiltration cell under nitrogen pressure (~20 psi) equipped with a 10 kDa molecular weight cutoff membrane (Millipore, Regenerated Cellulose membrane, diameter 63.5 mm, NMWL: 10,000). The protein was first concentrated to ~10 mL and then diluted to 150 mL with Tris buffer. This was again concentrated to less than 30 mL.

Purification was continued with Fast Protein Liquid Chromatography (NGC chromatography system, Biorad), equipped with a column loaded with Source 15Q (Q Sepharose Fast Flow, GE Healthcare) anion exchange resin. The method for FPLC source Q is shown in **Table S2**. The pH at all steps was set to 8.1. Percent B corresponds to the percent salt, where 100% is equivalent to 1 mM NaCl.

Table S2. FPLC Source 15Q method.

Step	Description	% B	Volume	Flow Rate
1.	Isocratic flow	0	12 mL	3 mL/min
2.	Load sample	n/a	Sample volume	2 mL/min
3.	Isocratic flow	0	10 mL	3 mL/min
4.	Gradient flow	0 to 4	12 mL	3 mL/min
5.	Isocratic flow	4	20 mL	3 mL/min
6.	Gradient flow	4 to 8	15 mL	3 mL/min
7.	Isocratic flow	8	20 mL	3 mL/min
8.	Gradient flow	8 to 15	15 mL	3 mL/min
9.	Isocratic flow	15	40 mL	3 mL/min
10.	Gradient flow	15 to 75	10 mL	3 mL/min
11.	Isocratic flow	100	20 mL	3 mL/min
12.	Isocratic flow	0	35 mL	3 mL/min

Protein was then collected from 40 mM, 80 mM or 150 mM NaCl and concentrated to 1 mL using a 10 kDa Centriprep centrifugal filter (Millipore, Regenerated Cellulose membrane, NMWL: 10,000). The concentrated sample was then loaded to the Fast Protein Liquid Chromatography (NGC chromatography system, Biorad), equipped with a column loaded with size exclusion chromatography (SEC) Superdex 75 Prep Grade resin (GE Healthcare). The method for FPLC SEC is shown in **Table S3**. At all steps, the pH was set to 8.1.

Table S3. FPLC SEC method.

Step	Description	% B	Volume	Flow Rate
1.	Load sample	0	4 mL	1 mL/min
2.	Isocratic flow	20	139 mL	1 mL/min

D. Extinction Coefficient Determination of hCRBPII Mutants

The absorption extinction coefficients (ε) for the various hCRBPII mutants were determined according to the method first described by Gill and von Hippel and were measured in triplicates (average is reported).[3] Extinction coefficients are listed in **Table S9**. The hCRBPII mutants designed previously were expressed in BL21(DE3)pLysS competent cells and the proteins were purified as described before.[2]

E. UV-Vis Measurements of hCRBPII/Chromophore Complexes

UV-Vis spectra were recorded with a Cary 300 Bio WinUV, Varian spectrophotometer. For all experiments, 20 μM protein was incubated with ligand (10 μM, 0.5 equiv) in PBS buffer and incubated at room temperature until Schiff base (SB) or protonated Schiff base (PSB) formation was complete. This was verified by UV-Vis.

F. pK_a Measurements of hCRBPII/Chromophores Complexes

For pK_a determination, protein (20 μM in PBS) was incubated with ligand (0.5 equiv) and incubated at room temperature until Schiff base (SB) or protonated Schiff base (PSB) formation was complete. This was verified by UV-Vis. The solution was then titrated with acid (with 1 M NaOAc, pH 4) or base (1 M NaOH) in ~0.3 pH units, and the absorption spectra were recorded at each point.

Absorbance change at λ_{max} was plotted as a function of pH. A curve fit, as previously described for bacteriorhodopsin, was applied for pK_a determination:

$$\Delta A = \frac{\Delta A_0}{(1 + 10^{pH - pK_a})} + constant$$

The two parameters are: ΔA_0, the total absorbance change of the PSB and pK_a, the midpoint of titration. It should be noted that a constant is included to account the deviation from zero absorbance intensity of the deprotonated PSB. In all cases, the reported pK_a is the average of a triplicate measurement.

G. Kinetic Measurements of hCRBPII/Chromophore PSB Formation

Half-life binding measurements ($t_{1/2}$) were determined under stoichiometric conditions (10 μM Q108K:K40L:T51V:T53S:R58W:Y19W:L117E (**M22**) and Thiofluor(**TD-IV**) in PBS buffer at 37 ˚C.[4] The increase in fluorescence intensity, indicative of binding and PSB formation, was recorded at 2 sec intervals, with excitation at 563 nm (1 nm slit width) and emission at 673 nm (12 nm slit width). The data was fit to a second-order rate equation derived as shown. The bound complex *C* is directly proportional to fluorescence intensity (cps), plotted vs. time (s). The maximum fluorescence intensity is adjusted to 10 μM, which is the final concentration of the complex, in order to fit the data shown below.

$$A + B \rightarrow C$$

Here, *A* = hCRBPII; *B* = **TD-IV**; and *C* = Bound Complex

$$\frac{d[C]}{dt} = [A][B]k_2 \qquad (\mathbf{1})$$

Initial concentrations of reactants $[A]_0 = [B]_0 = 10\mu M$

As the reaction continues, both *A* and *B* get consumed simultaneously in order to form a bound complex, *C*

$$\therefore [A] = [B] = [10\mu M - C]$$

From eq (**1**):

$$\frac{d[C]}{dt} = k_2[10 \times 10^{-6}M - C]^2$$

$$\frac{d[C]}{d[10 \times 10^{-6}M - C]^2} = k_2 dt$$

$$\int \frac{d[C]}{d[10 \times 10^{-6}M - C]^2} = \int k_2 dt$$

Here, $\frac{1}{[10 \times 10^{-6}M - C]} = k_2 t + m$; where m is a constant

$C = 10 \times 10^{-6}M - \frac{1}{[k_2 t + m]}$ (**2**)

Equation (**2**) is rewritten in kaleidaGraph as:

$$y = m_3 - \frac{1}{[m_2 m_0 + m_1]}$$

Where concentration of the limiting reactant $m_3 = 10 \times 10^{-6}M$;

$m_2 = k_2,\ m_0 = t$ and m_1 is a constant.

The half-life ($t_{1/2}$) of the reaction can be calculated as:

$$\therefore t_{1/2} = \frac{1}{k[A]_o}$$

This is used for the kinetic measurement in Figure S9.

H. Fluorescence Measurements

Fluorescence spectra were recorded using a Fluorolog®-3 spectrofluorometer (HORIBA, Ltd.). An entrance slit of 1 nm and exit slit of 12 nm was used for all measurements.

I. Quantum Yield Measurements

Absolute fluorescence quantum yields (Φ) were measured on a Quantaurus-QY (model C11347-11, Hamamatsu Photonics) equipped with a xenon light source, monochromator, integration sphere and a multichannel back-thinned CCD detector at room temperature. The reported values represent the average of triplicate measurements.

J. Cloning for Mammalian Expression Vectors

General Cloning Protocol

The DNA fragment was amplified using Phusion High-Fidelity DNA Polymerase (NEB, M0530) with the appropriate primers (see below for details). PCR conditions are specified in **Table S4** using a Bio-Rad iCycler thermal cycler. Four reactions were set up for each cloning, to ensure that enough amplified product was obtained.

Table S4. PCR cycling conditions for cloning.

	PCR Program	Time (min)
1x	98 °C	0:30
40x	98 °C	0:10
	55 °C	0:30
	72 °C	Extension time
1x	72 °C	10:00
1x	4 °C	10:00

Reactant	Volume
DNA template	100 ng (x μL)
Forward primer	20 pmol (y μL)
Reverse primer	20 pmol (z μL)
10 mM dNTP	1 μL
50 mM $MgCl_2$	1μL
DMSO	5 μL
5 x Phusion HF Reaction Buffer	10 μL
Phusion HF DNA Polymerase	0.5 μL
Nuclease-free water	50 μL– (x+y+z) –17.5 μL

The PCR amplified gene was purified by Wizard® SV Gel and PCR Clean-Up System (Promega) from 1% agarose gel in amount of 20-50 ng/μL. The product was digested with the proper enzymes and ligated to a similarly prepared plasmid (50 ng/μL). Ligation between the insert fragment and plasmid was performed with 30 ng of plasmid and 90 ng of insert using T4 DNA Ligase (New England BioLabs). The ligated product was transformed into *E. coli* XL1-blue competent cells (Agilent) and grown on LB-agar plates supplemented with antibiotics (100 μg/mL ampicillin, 7.5 μg/mL tetracycline) at 37 ºC for 18 hours. Colonies were inoculated in LB medium (15 mL) with proper antibiotics

(100 μg/mL ampicillin, 7.5 μg/mL tetracycline) and incubated at 37 ºC while shaking, for 10 hours. DNA purification was performed using Promega Wizard® Plus SV Miniprep DNA purification kit (A1330) following the manufacturer's protocol. The DNA sequence was verified with the corresponding sequencing primers by the Research Technology Support Facility at Michigan State University.

CMV end_Seq: 5'-GGTCTATATAAGCAGAGCTGGTTTAG-3'

midGFP: 5'-CGTGCTGCTGCCCGACAACC-3'

Preparation of Plasmids

The plasmids described previously HindIII-EGFP-NotI-hCRBPII$_{tetra}$-Stop-BamHI (Plasmid1) in pFlag-CMV2, HindIII-EGFP-NotI-hCRBPII$_{tetra}$Q108L-Stop-BamHI (Plasmid2) in pFlag-CMV2, and HindIII-EGFP-NotI-hCRBPII$_{nona}$-Stop-BamHI (Plasmid3) in pFlag-CMV2 were used in constructing the plasmids discussed in this paper.[5] Plasmids used in this study were constructed by amplifying the hCRBPII gene of interest from pET-17b, Q108K:K40L:T51V:T53S:R58W:Y19W:L117E. Forward and reverse primers used were 5'-CGGCGGCCGCATGACGAGGGAC-3' and 5'-CGGAATTCGCACTTCTTTTTGAACACTTG-3' (see **Table S4** for protocol, extension time = 15 sec). The purified product was then inserted between NotI and EcoRI of Plasmid4. The product was sequenced with the primers CMVend_Seq and midGFP.

K. DNA Sequences of Mammalian Plasmids

Plasmid1: HindIII-EGFP-NotI-Q108K:K40L:T51V:T53S:R58W:Y19W:L117E-BamHI-RVASL-Stop (pFlag-CMV2)

AAGCTTATGGTGAGCAAGGGCGAGGAGCTGTTCACCGGGGTGGTGCCCATCCTG
GTCGAGCTGGACGGCGACGTAAACGGCCACAAGTTCAGCGTGTCCGGCGAGGGC
GAGGGCGATGCCACCTACGGCAAGCTGACCCTGAAGTTCATCTGCACCACCGGCA
AGCTGCCCGTGCCCTGGCCCACCCTCGTGACCACCCTGACCTACGGCGTGCAGT
GCTTCAGCCGCTACCCCGACCACATGAAGCAGCACGACTTCTTCAAGTCCGCCAT
GCCCGAAGGCTACGTCCAGGAGCGCACCATCTTCTTCAAGGACGACGGCAACTAC
AAGACCCGCGCCGAGGTGAAGTTCGAGGGCGACACCCTGGTGAACCGCATCGAG
CTGAAGGGCATCGACTTCAAGGAGGACGGCAACATCCTGGGGCACAAGCTGGAG
TACAACTACAACAGCCACAACGTCTATATCATGGCCGACAAGCAGAAGAACGGCAT
CAAGGTGAACTTCAAGATCCGCCACAACATCGAGGACGGCAGCGTGCAGCTCGCC
GACCACTACCAGCAGAACACCCCCATCGGCGACGGCCCCGTGCTGCTGCCCGAC
AACCACTACCTGAGCACCCAGTCCGCCCTGAGCAAAGACCCCAACGAGAAGCGC
GATCACATGGTCCTGCTGGAGTTCGTGACCGCCGCCGGGATCACTCTCGGCATGG
ACGAGCTGTACAAGTCCGGAGCCGCTGCAGGAGGCGGCCGCATGACGAGGGACC
AGAATGGAACCTGGGAGATGGAGAGTAATGAAAACTTTGAGGGCTGGATGAAGGC
CCTGGATATTGATTTTGCCACCCGCAAGATTGCAGTACGTCTCACTCAGACGCTGG
TTATTGATCAAGATGGTGATAACTTCAAGGTAAAAAGCACTAGCACATTCTGGAACT
ATGATGTGGATTTCACTGTTGGAGTAGAGTTTGACGAGTACACAAAGAGCCTGGAT
AACCGGCATGTTAAGGCACTGGTCACCTGGGAAGGTGATGTCCTTGTGTGTGTGC
AAAAGGGGGAGAAGGAGAACCGCGGCTGGAAGAAGTGGATTGAGGGGGACAAGC
TGTACGAGGAGCTGACCTGTGGTGACCAGGTGTGCCGTCAAGTGTTCAAAAAGAA
GTGCGGATCCCGGGTGGCATCCCTGTGA

Plasmid2: HindIII-EGFP-NotI-Q108K:K40L:T51V:T53C:R58W:T29L:A33W:Q4F:L117E-Stop-BamHI (pFlag-CMV2)

AAGCTTATGGTGAGCAAGGGCGAGGAGCTGTTCACCGGGGTGGTGCCCATCCTG
GTCGAGCTGGACGGCGACGTAAACGGCCACAAGTTCAGCGTGTCCGGCGAGGGC
GAGGGCGATGCCACCTACGGCAAGCTGACCCTGAAGTTCATCTGCACCACCGGCA
AGCTGCCCGTGCCCTGGCCCACCCTCGTGACCACCCTGACCTACGGCGTGCAGT
GCTTCAGCCGCTACCCCGACCACATGAAGCAGCACGACTTCTTCAAGTCCGCCAT
GCCCGAAGGCTACGTCCAGGAGCGCACCATCTTCTTCAAGGACGACGGCAACTAC
AAGACCCGCGCCGAGGTGAAGTTCGAGGGCGACACCCTGGTGAACCGCATCGAG
CTGAAGGGCATCGACTTCAAGGAGGACGGCAACATCCTGGGGCACAAGCTGGAG
TACAACTACAACAGCCACAACGTCTATATCATGGCCGACAAGCAGAAGAACGGCAT
CAAGGTGAACTTCAAGATCCGCCACAACATCGAGGACGGCAGCGTGCAGCTCGCC
GACCACTACCAGCAGAACACCCCCATCGGCGACGGCCCCGTGCTGCTGCCCGAC
AACCACTACCTGAGCACCCAGTCCGCCCTGAGCAAAGACCCCAACGAGAAGCGC
GATCACATGGTCCTGCTGGAGTTCGTGACCGCCGCCGGGATCACTCTCGGCATGG
ACGAGCTGTACAAGTCCGGAGCCGCTGCAGGAGGCGGCCGCATGACGAGGGACT
TCAATGGAACCTGGGAGATGGAGAGTAATGAAAACTTTGAGGGCTACATGAAGGC
CCTGGATATTGATTTTGCCCTGCGCAAGATTTGGGTACGTCTCACTCAGACGCTGG
TTATTGATCAAGATGGTGATAACTTCAAGGTAAAATGCACTAGCACATTCTGGAACT
ATGATGTGGATTTCACTGTTGGAGTAGAGTTTGACGAGTACACAAAGAGCCTGGAT
AACCGGCATGTTAAGGCACTGGTCACCTGGGAAGGTGATGTCCTTGTGTGTGTGC
AAAAGGGGGAGAAGGAGAACCGCGGCTGGAAGAAGTGGATTGAGGGGGACAAGC

TGTACGAGGAGCTGACCTGTGGTGACCAGGTGTGCCGTCAAGTGTTCAAAAAGAA

GTGAGGATC

L. Mammalian Cell Culture

HeLa cell lines were cultured in Dulbecco's Modified Eagle medium (DMEM, supplemented with phenol red, 4.5 g/L Dglucose, L-glutamine and 110 mg/L Sodium Pyruvate; purchased from Sigma-Aldrich) supplemented with 10% (v/v) Fetal Bovine Serum (FBS, BioWest) and 1x Penicillin-Streptomycin-Glutamine (PSG; purchased from GIBCO) at 37 °C within a 5% CO_2 and 10% O_2 atmosphere. For microscopic imaging, cells were seeded on an ibidi 1 µ-Slide 8 well ibiTreat plate. After approximately 12 h the cells were transiently transfected using Genjet Ver. II (purchased from SignaGen) according to the manufacturer's protocol. Following transfection (after 48 h), the media was removed, and the cells were incubated with a media containing the fluorophore. To prepare the fluorophore solution, a stock solution in DMSO (~0.003 M) was warmed to room temperature. The exact concentration of the fluorophore stock solution was determined using UV-Vis (using the associated extinction coefficient). The stock solution was then diluted to the specified concentration with pre-heated (37 °C) DMEM. After the indicated fluorophore incubation period, the cells were washed two times with Dulbecco's Phosphate Buffered Saline (DPBS, supplemented with calcium chloride and magnesium chloride; purchased from Sigma Aldrich) an incubated in RPMI-1640 medium (without phenol red) for imaging immediately.

M. General Confocal Imaging Method

Cell microscopy was performed using an inverted laser scanning confocal microscope (LSM510Meta, Carl Zeiss, Jena, Germany) equipped with diode, argon and HeNe lasers. A 40x oil-immersed objective was used.

ECFP was imaged using 458 nm excitation, 458 nm primary dichroic, 515 nm secondary dichroic and BP 475–525 nm emission. hCRBPII/ThioFluor was imaged using multiple settings - 594 nm excitation, 594 nm primary dichroic, 545 nm secondary dichroic and LP 615 nm emission; DICII images were also collected. Kalman averaging 8 was applied in all confocal images. Fluorescence in each experiment was normalized to the same intensity adjusting the gain and amplifier offset. All images are pseudo-colored.

N. Spectroscopic Tables of TD-1V and its Bound Form with Mutants.

Table S5. Spectroscopic characterization of **TD-1V** in various solvents.

Solvent	λ_{abs}	λ_{em}	Stokes shift	ε ($M^{-1}cm^{-1}$)	Φ^a
Toluene	422	516	94	32,149	0.02
Ethyl acetate	414	558	144	28,939	0.11
Dimethyl sulfoxide	437	623	186	26,891	0.53
Ethanol	430	654	224	27,021	0.06
PBS buffer	393	-	-	8,842	0.00

[a]Absolute quantum yield was measured on a Quantaurus-QY.

Table S6. Spectroscopic properties of **TD-1V**-PSB with *n*-butyl amine.

Solvent	λ_{abs} (nm)	λ_{em} (nm)	Stokes Shift (nm)	Φ^a
PBS buffer	464	-	-	-
Ethanol	521	689	168	0.02
Tetrahydrofuran	494	674	180	0.17
Formamide	525	707	182	0.01
Acetone	493	687	194	0.03
Dimethyl sulfoxide	512	716	204	0.02

[a]Absolute quantum yield was measured on a Quantaurus-QY.

Table S7. List of Mutants that did not Express Solubly.

Entry	Mutants
1	Q108K:K40E:R58L:Q38F:Q4F:W8F:W88F
2	Q108K:K40L:T53A:R58W:Q38F:Q4F:Y19W:I42E
3	Q108K:K40E:R58L:Q38F:Q4F:W88F:W109F
4	Q108K:K40E:R58L:Q38F:Q4F:W8F:W106F
5	Q108K:K40E:R58L:Q38F:Q4F:W88F:W106F
6	Q108K:K40E:R58L:Q38F:Q4F:W8F:W109F
7	Q108K:K40E:T53A:R58L:Q38F:Q4F:I42E
8	Q108K:K40E:T53A:R58L:Q38F:Q4F:W8E
9	Q108K:K40E:T53A:R58L:Q38F:Q4F:L115E
10	Q108K:K40E:T53A:R58L:Q38F:Q4F:F130E
11	Q108K:K40L:T53A:R58L:Q38F:Q4E
12	Q108K:K40L:L115E
13	Q108K:K40D:T51V:T53S:R58W:Y19W:L117E
14	Q108K:K40D:T51V:T53S:R58W:Y19W:V62E
15	Q108K:K40E:T53A:R58L:Q38L
16	Q108K:K40D:T53A:R58L:Q38F
17	Q108K:K40N:T53S:V62E
18	Q108K:K40L:T53S:V62E:L77S
19	Q108K:K40L:T53S:V62N:F64E
20	Q108K:K40L:T51V:T53S:R58W:Y19W:L77S

Table S8. Results of introducing acidic residues to blue shift wavelength.

Mutant	Protein	λ_{abs} (nm)	λ_{em} (nm)	Φ^a
**M25**	KL:T53A:R58L:Q38F	577	671	0.14
**M26**	KL:T53A:R58L:Q38F:Q4F	616	691	0.10
**M27**	Q108K:K40E:T53A:R58L:Q38F:Q4F	575	671	0.15
**M28**	Q108K:K40D:T53A:R58Y:Q38F:Q4F	551	641	0.26
**M39**	Q108K:K40L:T51D:T53A:R58Y:Q38F:Q4F	576	665	0.25
**M30**	Q108K:K40L:T53A:R58Y:Q38F:Q4F:L117E	510	653	0.20
**M31**	Q108K:K40L:T53A:R58Y:Q38F:Q4F:V62E	584	656	0.15
**M32**	Q108K:K40L:T51D:T53A:R58Y:Q38F:Q4F:L117E	501	613	0.49
**M33**	Q108K:K40D:T53A:R58Y:Q38F:Q4F:L117E	505	653	0.13
**M34**	Q108K:K40E:T53A:R58L:Q38F:Q4F:V62E	521	656	0.24

Table S9. Extinction coefficients of hCRBPII mutants.

Mutant	Protein	$\varepsilon_{exp(280\ nm)}$
**M1**	Q108K:K40L	27,000
**M2**	Q108K:K40L:T51S	33,000
**M3**	Q108K:K40L:T51V	29,000
**M4**	Q108K:K40L:T53A	29,000
**M5**	Q108K:K40L:T53S	28,000
**M6**	Q108K:K40L:Q38F	30,000
**M7**	Q108K:K40L:Q4F	30,000
**M8**	Q108K:K40L:T51V:T53S	31,000
**M9**	Q108K:K40L:T51V:T53S:R58F	29,000
**M10**	Q108K:K40L:T51V:T53S:R58Y	29,000
**M11**	Q108K:K40L:T51V:T53S:R58W	34,000
**M12**	Q108K:K40L:T51V:T53S:R58H	28,000
**M13**	Q108K:K40L:T51V:T53S:R58H:Y19W	34,000
**M14**	Q108K:K40L:T51V:T53S:R58W:Y19W	37,000
**M15**	Q108K:K40L:T51V:T53S:R58W:Y19W:A33W	43,000
**M16**	Q108K:K40L:T51V:T53S:R58W:Y19W:L77W	45,000
**M17**	Q108K:K40L:T51V:T53S:R58W:Y19W:F16Y	45,000
**M18**	Q108K:K40L:T51V:T53S:R58W:Y19W:A33W:Q4W	52,000
**M19**	Q108K:K40L:T51V:T53S:R58W:Y19W:A33W:Y60W	49,000
**M20**	Q108K:K40L:T51V:T53S:R58W:Y19W:A33W:S76W	52,000
**M21**	Q108K:K40L:T51V:T53S:R58W:Y19W:A33W:L119W	50,000
**M22**	Q108K:K40L:T51V:T53S:R58W:Y19W:L117E	36,000
**M23**	Q108K:K40L:T51V:T53S:R58W:Y19W:L117D	38,000
**M24**	Q108K:K40L:T53A:R58L:Q38F	29,000
**M25**	Q108K:K40L:T53A:R58L:Q38F:Q4F	31,000
**M26**	Q108K:K40E:T53A:R58L:Q38F:Q4F	29,000
**M27**	Q108K:K40D:T53A:R58Y:Q38F:Q4F	26,000
**M28**	Q108K:K40L:T51D:T53A:R58Y:Q38F:Q4F	30,000
**M29**	Q108K:K40L:T53A:R58Y:Q38F:Q4F:L117E	29,000
**M30**	Q108K:K40L:T53A:R58Y:Q38F:Q4F:V62E	28,000
**M31**	Q108K:K40L:T51D:T53A:R58Y:Q38F:Q4F:L117E	30,000
**M32**	Q108K:K40D:T53A:R58Y:Q38F:Q4F:L117E	33,000
**M34**	Q108K:K40E:T53A:R58L:Q38F:Q4F:V62E	25,000

O. Supporting Information Figures

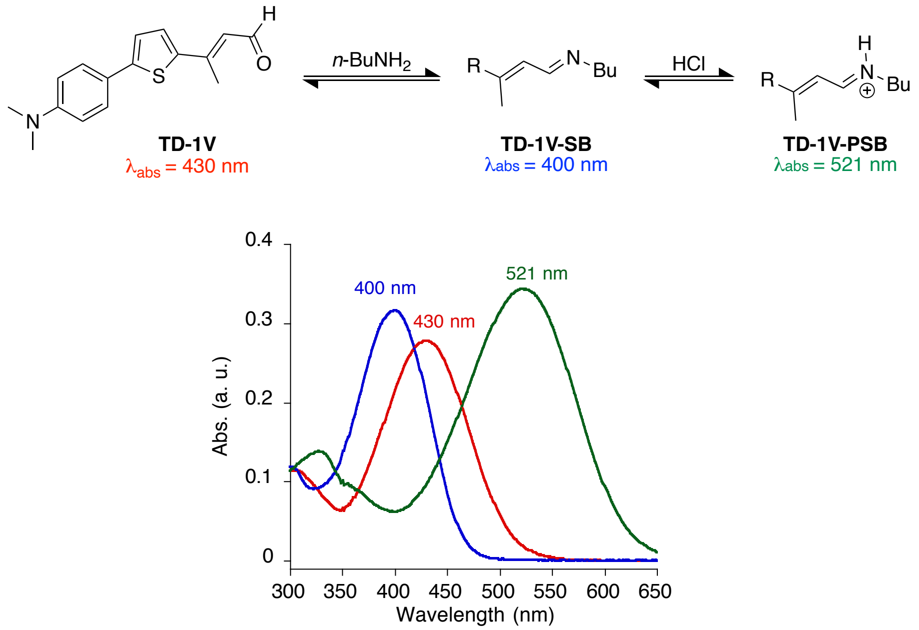

Figure S1. Formation of the protonated Schiff base (PSB) of **TD-1V** with *n*-butyl amine in ethanol.

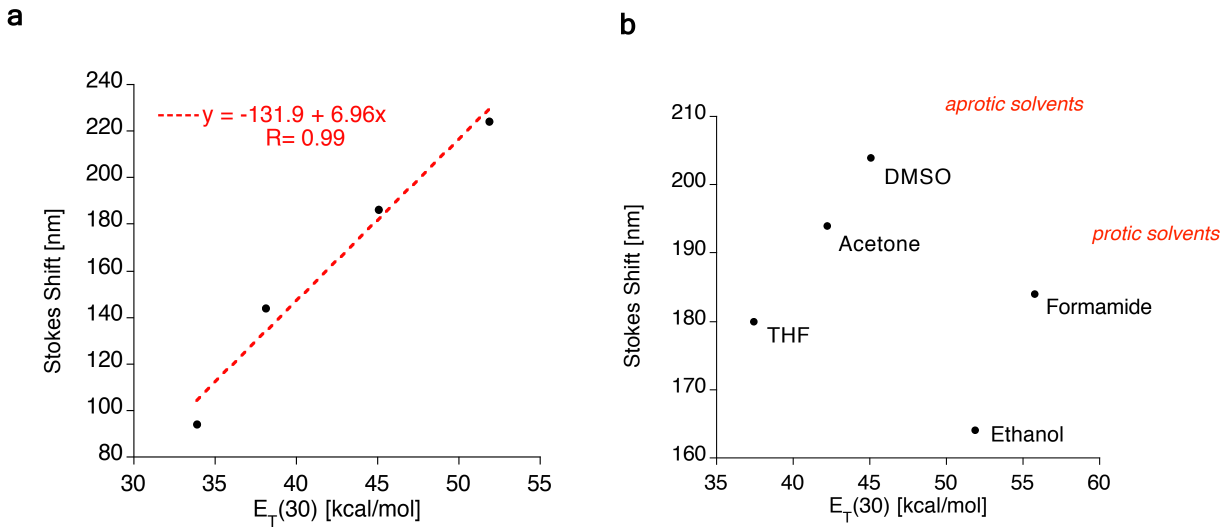

Figure S2. a. Stokes shift in different solvents versus the $E_T(30)$ value indicates that **TD-1V** is solvatochromic. The extinction coefficients of **TD-1V** in toluene, ethyl acetate, dimethyl; b. Stokes shift in different solvents versus the $E_T(30)$ value indicates that **TD-1V**-PSB is not solvatochromic. A correlation is observed in protic and aprotic solvents independently.

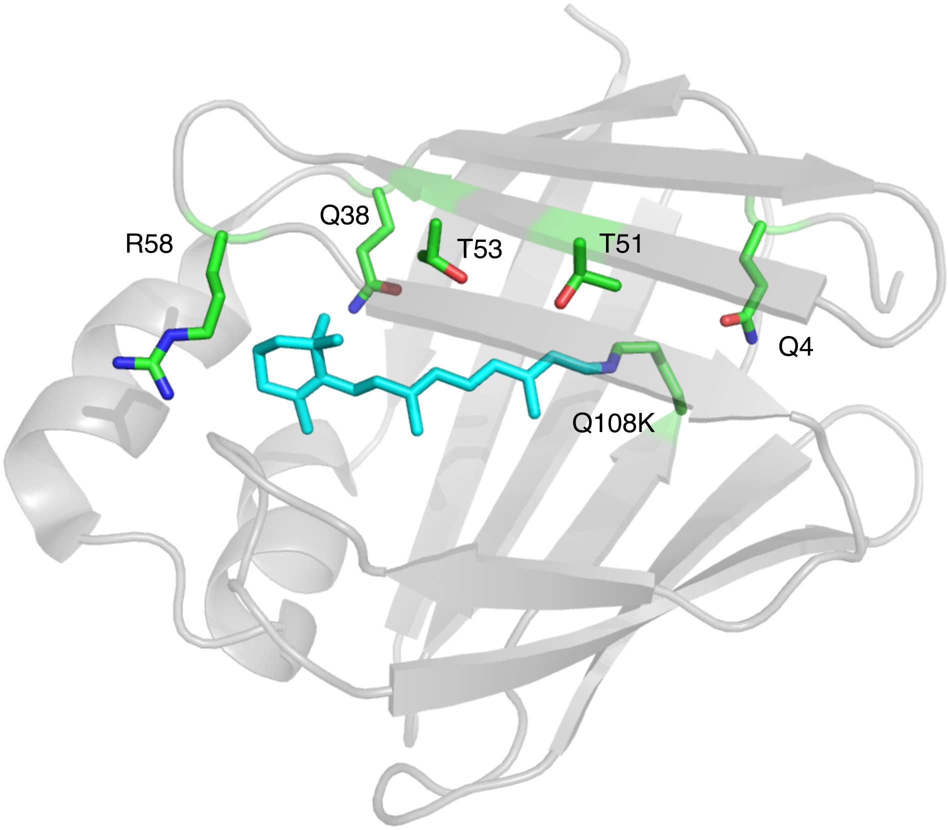

Figure S3. Polar residues mutated in order to remove polarity from the binding pocket. Coordinates obtained from PDB 4EXZ (hCRBPII-Q108K:K40L/retinal).

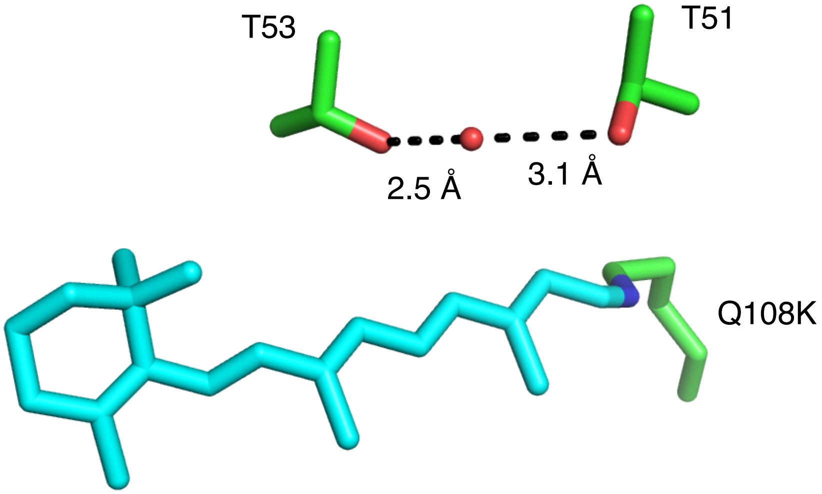

Figure S4. Water mediated hydrogen bonding between T51 and T53. Coordinates obtained from PDB 4EXZ (hCRBPII-Q108K:K40L/retinal).

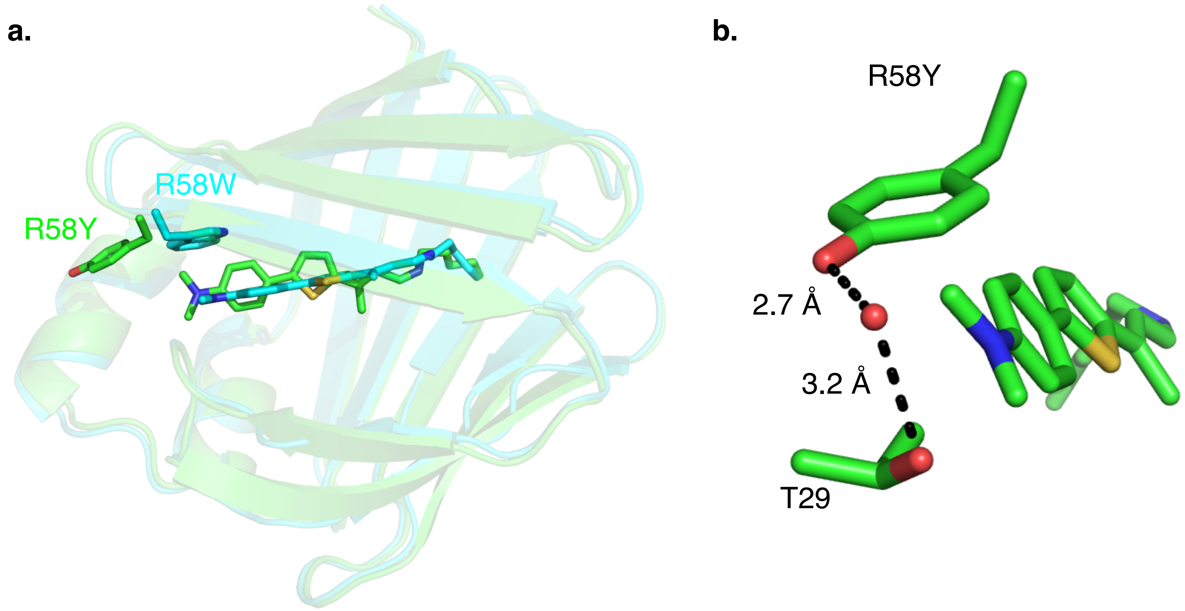

Figure S5. Crystal structure of Q108K:K40L:T51V:T53S:R58W(**M11**)/**TD-1V** (cyan) overlaid with Q108K:K40L:T51V:T53S:R58Y(**M10)**/**TD-1V** (green); **b.** Water mediated hydrogen bonding between R58Y and T29.

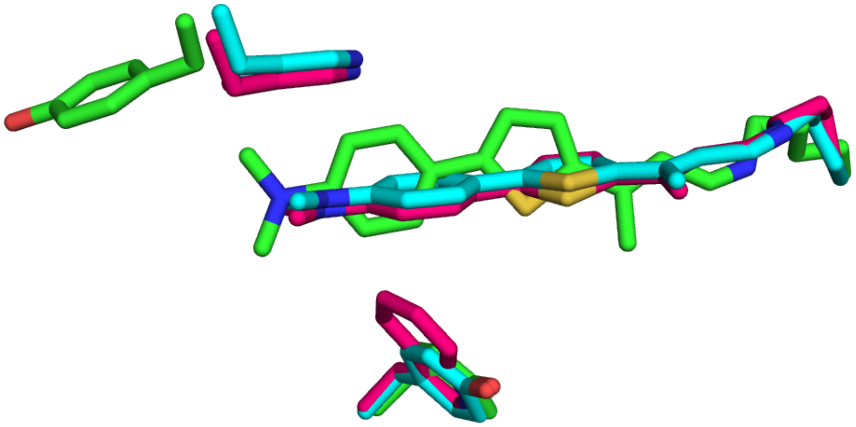

Figure S6. Crystal structure of Q108K:K40L:T51V:T53S:R58Y/**TD-1V** (**M10,** green) overlaid with Q108K:K40L:T51V:T53S:R58W/ **TD-1V** (**M11**, cyan) and Q108K:K40L:T53A:Y19W:R58W/**TD-1V**(**M14**, pink). Note the 90° rotation of **TD-IV** in **M10** relative to **M11** and **M14** caused by the cation-π interaction with Trp58 in the latter two structures.

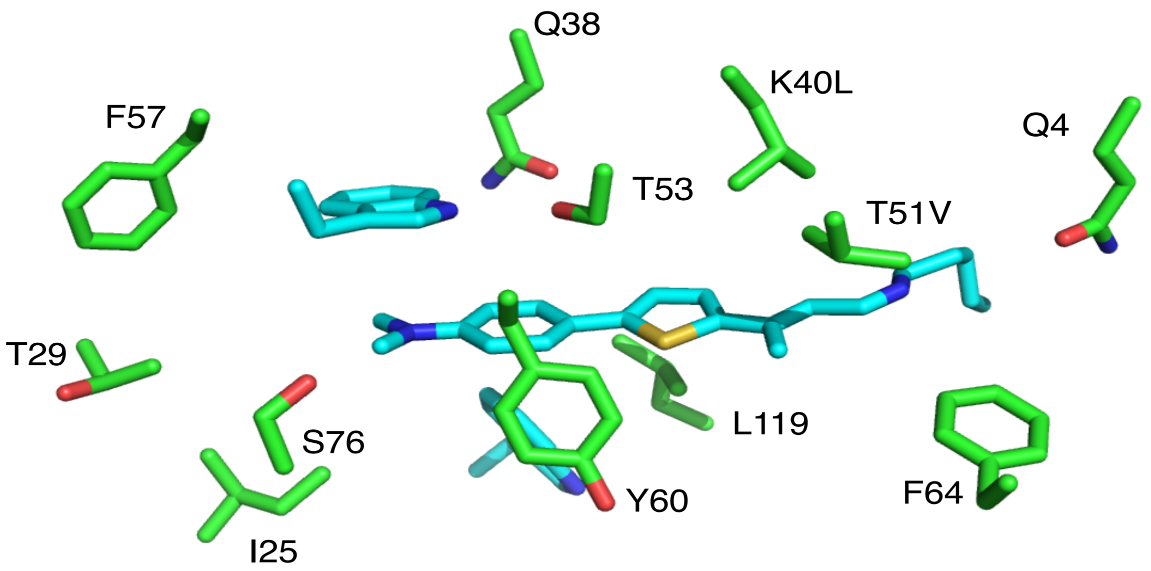

Figure S7. Residues at which tryptophan was introduced in an attempt to red shift wavelength are shown in green. Crystal structure is of Q108K:K40L:T51V:T53S:R58W:Y19W(**M14**)/**TD-IV**.

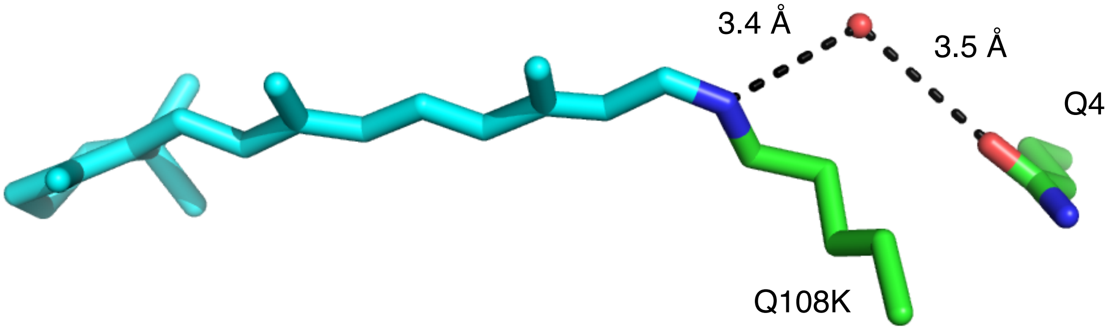

Figure S8. Water mediated hydrogen bonding between Q4 and the hCRBPII/retinal iminium. Coordinates obtained from PDB 4EFG (hCRBPII-Q108K:K40L:T51V:T53C:R58W:T29L:Y19W/retinal).

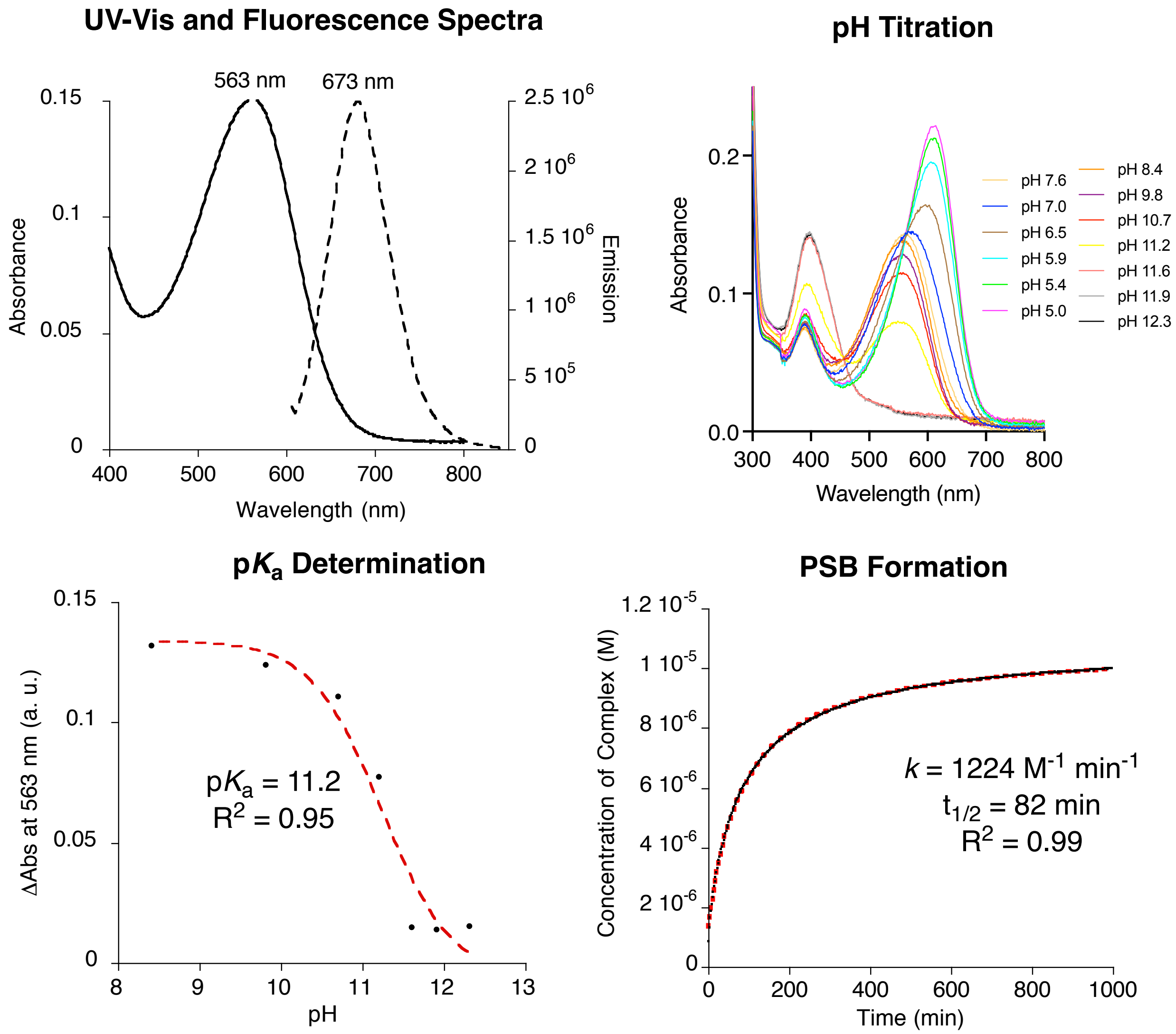

Figure S9. UV-Vis, fluorescence, pH titration, pK_a determination, and rate of PSB formation for Q108K:K40L:T51V:T53S:R58W:Y19W:L117E **(M22)**/**TD-1V** complex.

P. Protein Crystallization and Data Collection

The proteins were concentrated between 6 and 8 mg/mL in a buffer A (10 mM Tris, 150 mM NaCl, pH = 8). Prior to crystallization, the protein was incubated with four equivalent chromophores. Crystals were grown through the hanging drop vapor diffusion method by adding 1 μL of protein solution and 1 μL of crystallization solution in the drop and 1 mL of crystallization solution in the reservoir. All of the crystallization plates were wrapped in aluminum foil to protect the chromophore from light damage.

The crystallization solution contained 25-30% PEG 4000, 0.1 M sodium acetate, pH=4.0-4.8, 0.1 M ammonium acetate. The cryoprotectant solution (30% PEG4000, 0.1 M sodium acetate pH = 4.5, 0.1 M ammonium acetate with 15% glycerol) was employed for soaking and flash freezing crystals in loops in liquid nitrogen. The crystals were stored in a liquid nitrogen Dewar prior to data collection.

The diffraction data were collected at the Advanced Photon Source (APS) (Argonne National Laboratory IL) LS-CAT, (sector 21-ID-D, F, G) using Dectris 9M Eiger and Rayonix MX300 detectors, ~1.00Å wavelength radiation at 100K. The initial diffraction data were indexed, processed, and scaled using the HKL2000 software package. The structures were solved by molecular replacement using PHASER in PHENIX and hCRBPII (PDB entry 2RCQ) as a search model. The initial electron density map was generated by Phaser-MR in PHENIX. Model rebuilding, placement of water molecules etc. were performed using COOT. The structures were refined through the PHENIX program packages. The chromophore was created using the Coot program and restraints was generated by PHENIX.

Q. Crystallographic Data and Refinement Statistics

	Q108K:K40L :T53A:R58F	Q108K:K40L:T51V :T53S:R58Y	Q108K:K40L:T51V :T53S:R58W	Q108K:K40L:T51V :T53S:R58W:Y19W	Q108K:K40L:T51V:T53S :R58W:Y19W:L117E
**Resolution Range (Å)**	27.4 - 1.47 (1.51 - 1.41)	29.2 - 1.34 (1.37 - 1.34)	27.36 - 1.499 (1.553 - 1.499)	29.60 - 1.220 (1.25 - 1.220)	29.55 - 1.24 (1.27 - 1.23)
**Space group**	P1	P1	P1	P1	P1
**a (Å)**	36.51	32.29	29.889	29.32	29.40
**b (Å)**	55.16	35.25	36.209	36.43	36.39
**c (Å)**	68.87	64.87	64.081	64.08	64.27
**a (°)**	109.94	92.51	90.32	89.74	89.84
**b (°)**	98.49	90.42	91.875	89.36	89.66
**γ (°)**	101.41	111.72	113.625	66.38	66.15
**Molecules per Asymmetric Unit**	4	2	2	2	2
**Total reflection**	597246	268817	415713	544327	623264
**Unique Reflection**	82805	47165	39448	72294	69070
**Completeness (%)**	95.71 (91.0)	79.62 (94.6)	96.4 (94.4)	93.53 (90.0)	94.8 (89.0)
**Average I/s**	32.9 (2.01)	43.37 (1.64)	41.1 (3.2)	44.63 (6.9)	31.4 (1.47)
**R_{work}/R_{free} (%)**	23.3/26.3	23.8/28.2	20.7/23.8	17.9/18.3	17.3/18.5
**RMSD from ideal values**					
Bond Length (Å)	0.008	0.013	0.017	0.01	0.007
Bond Angle	1.249	1.56	1.79	1.58	1.30
Average B factor	15.89	20.58	21.22	12.07	13.66
Number of water molecules	513	284	149	321	211
PDB IDs	8VZX	8VZY	8W02	8VZZ	8W00

R. References

1. Santos, E. M.; Sheng, W.; Esmatpour Salmani, R.; Tahmasebi Nick, S.; Ghanbarpour, A.; Gholami, H.; Vasileiou, C.; Geiger, J. H.; Borhan, B., Design of Large Stokes Shift Fluorescent Proteins Based on Excited State Proton Transfer of an Engineered Photobase. *J. Am. Chem. Soc.* **2021**, *143*, 15091-15102.
2. Wang, W.; Nossoni, Z.; Berbasova, T.; Watson, C. T.; Yapici, I.; Lee, K. S. S.; Vasileiou, C.; Geiger, J. H.; Borhan, B., Tuning the Electronic Absorption of Protein-Embedded All-trans-Retinal. *Science* **2012**, *338*, 1340-1343.
3. Gill, S. C.; von Hippel, P. H., Calculation of protein extinction coefficients from amino acid sequence data. *Anal. Biochem.* **1989**, *182*, 319-26.
4. Yapici, I.; Lee, K. S. S.; Berbasova, T.; Nosrati, M.; Jia, X.; Vasileiou, C.; Wang, W.; Santos, E. M.; Geiger, J. H.; Borhan, B., "Turn-On" Protein Fluorescence: In Situ Formation of Cyanine Dyes. *J. Am. Chem. Soc.* **2015**, *137*, 1073-1080.
5. Santos, E. M.; Berbasova, T.; Wang, W.; Salmani, R. E.; Sheng, W.; Vasileiou, C.; Geiger, J. H.; Borhan, B., Engineering of a Red Fluorogenic Protein/Merocyanine Complex for Live-Cell Imaging. *ChemBioChem* **2020**, *21*, 723-729.
